# Role of Glutathione S Transferase Polymorphism in COPD with Special Reference to Peoples Living in the Vicinity of the Open Cast Coal Mine of Assam

**DOI:** 10.1371/journal.pone.0096739

**Published:** 2014-05-08

**Authors:** Tapan Dey, Kabita Gogoi, Bala Gopalan Unni, Munmi Kalita, Moonmee Bharadwaz, Minakshi Bhattacharjee, Pranab Kumar Boruah, Thaneswar Bora, Dibyajyoti Ozah, Manoj Kalita

**Affiliations:** 1 Biotechnology Division, Council of Scientific & Industrial Research (CSIR) - North-East Institute of Science and Technology, Jorhat, Assam, India; 2 Biotechnology Department, University of Science and Technology Meghalaya (USTM), 9th Mile, Meghalaya, India; Institut Jacques Monod, France

## Abstract

**Background:**

COPD may develop due to variation in the functioning of antioxidants along with smoking and environmental factors in genetically susceptible individuals. Since there are different views about the antioxidants responsible for detoxifying xenobiotic compound in the human body whose functional variation may lead to obstructive disease, this associative study has been taken up between GST gene polymorphism and COPD in populations exposed to coal dusts.

**Methods:**

Genotypes of the 70 COPD patients and 85 non COPD patients were determined by PCR based methods followed by multiplex PCR of GSTT1 and GSTM1 genes taking albumin gene as a control. Suspended particulate analyses were determined through the Respirable Dust sampler along with the FTIR analysis of the dust samples from the glass microfiber filters.

**Results:**

Dust sampling analysis reveals higher level of respirable suspended particulate matter, non respirable particulate matter, SO_2_ and NO_2_ present in air of the study site. FTIR analysis also suggests a higher concentration of organic silicone and aliphatic C-F compounds present in air of the study site and when spirometry was done, low lung function was observed among most of the subjects. GSTM1 null type was significantly associated with low lung function in smoker groups and the presence of at least one active allele (either GSTM1/GSTT1) seemed to have a protective role in the development of COPD.

**Conclusions:**

GSTM1 (null genotype) appeared to be a risk factor for lower lung function in smokers living in the vicinity of coal mines. Apart from polluted environment and genetic susceptibility, mixed coal dust exposure rich in organic silicone and aliphatic C-F compounds also appears to be a factor for the low lung function.

## Introduction

Globally, Chronic Obstructive Pulmonary Disease (COPD) is one of the leading causes of mortality and by 2020 it is expected to rise to the third position as a cause of death and in fifth position as the cause of disability adjusted life years (DALYs) as per projections made in the Global Burden of Disease study (GBDS) [1].

Toxic particles and gases that are present in the atmosphere are likely to be inhaled or often self-administered through cigarette smoke, causing lung injury. However, contamination of atmosphere from anthropogenic sources such as coal mining, industrial sources as well as local conditions generated either in the home or workplace makes a significant contribution to the development of COPD. The relative prevalence and severity of mining related occupational lung diseases are a function of the commodities mined, airborne hazard exposure levels, and co-existing illnesses or environmental conditions and lifestyle. Chronic Obstructive Pulmonary Disease (COPD) is thought to be the result of environmental triggered in genetically susceptible individuals. Alpha 1 Antitrypsin is the only known genetic cause of COPD. Bhattacharjee et al. earlier studied the polymorphism of α 1-antitrypsin gene in the population of the same area where we have taken up the study [2]–[3]. COPD is the consequence of an abnormal inflammatory response due to inhalation of noxious agents such as cigarette smoking, occupational or environmental exposure. In fact only a portion (10–20%) of heavy smokers develops a clinically detectable disease [4]–[5]. Antioxidants and other less well understood protective mechanism may also be important in preserving normal lung function in the face of a lifetime exposure to potentially injurious environmental factors. Oxidative injury may also play an important role in the pathogenesis of COPD, [6]. Such injury, resulting from an imbalance between free radicals and protective mechanisms, can alter the conformation of protease inhibitors and reparative enzymes, injure cell membranes, and may result in mutagenesis. Free radicals appear in the lungs through inhalation from the environment or by its release from inflammatory cells inside the body. Genetically controlled antioxidant defence systems may also play an important role in determining susceptibility, both to free radicals released by inflammatory cells and to oxidants inhaled from the environment. The lung possesses several enzymatic scavengers including glutathione which are under genetic control. The observation that the enzymatic antioxidants are under genetic control and the allelic variations of these antioxidants alter their abilities to reduce free radicals, [7]–[8] suggests that genetic factors may place some individuals at greater risk for oxidant injury. The glutathione system is the major antioxidant mechanism in the airways. The increased oxidative stress in the airways of COPD patients may play an important pathophysiological role in the disease development by amplifying the inflammatory response in COPD.

COPD is characterized by progressive development of airflow limitation that is not fully reversible. It encompasses chronic obstructive bronchitis, with obstruction of small airways, and emphysema with enlargement of air spaces and destruction of lung parenchyma, loss of lung elasticity and closure of small airways [9].

The Glutathione S Transferase's (GST's), a super family of enzymes consisting of alpha, mu, pi, theta, kappa, zeta, sigma, omega and delta families, are critical in the conversion of many reactive electrophilic compounds to less reactive metabolites which are excreted as glutathione conjugates. Several common variants of GST's have been well characterized and are associated with certain respiratory diseases, [10]. These genes are expressed in the respiratory tract and have common functional variant alleles that result in either a total absence or a substantial change in enzyme activity. Thus the study has taken up to evaluate the role of polymorphism of the detoxifying enzyme GST in the pathogenesis of COPD with respect to polluted environment where the population is staying.

## Methods

The study was conducted at CSIR-North East Institute of Science and Technology, Jorhat, Assam after ethical clearance from the Institutional Ethics Committee, NEIST, Jorhat.

### Air Analysis

Air sampling was done with the help of Respirable Dust Sampler (Envirotech Model APM 460 DXNL) and data's were calculated for Respirable suspended particulate matter (RSPM) and Suspended Particulate Matter (SPM). Determination of SO_2_ concentration present in ambient air was done with the help of modified West and Gaeke Method and NO_2_ concentration was determined by Sodium Arsenite Method (as per manufacturer's instructions by the Central Pollution Control Board, India). Air analysis was carried out during the period January 2012 to January 2013.

### FTIR analysis of the dust samples

Organic extracts of dust samples of air were collected by the glass microfiber filters of size 1.6 µm. These extracts were then analysed through the FTIR system 2000 (Perkin Elmer, USA, ANC-1). The dust samples taken from the non-coal mine site i.e. around the research institute served as a control.

### Survey at the study site

A survey was conducted in the vicinity of the open-cast coal mine area of Assam. Questions regarding smoking habits, age, gender, socioeconomic status, occupation, environment etc. were included in the questionnaire with special concern on the epidemiology of GST gene.

Among 575 subjects were surveyed from the study site and the overall disease symptoms reported were fever, frequent cough and cold, weakness, and high blood pressure. Among the 575 subjects surveyed only 155 subjects visited the nearest mini PHC at Ledo during the period January 2012 to January 2013. Of these, about 45.16% (70) were suffering from COPD related respiratory symptoms. Baseline characteristics of all the study subjects were shown in Table 1.

**Table 1:** Baseline and clinical characteristics of the study subjects.

Variables Study population	Smokers with COPD (n = 38)	Non-smokers with COPD (n = 32)	^Healthy non^ Smokers (n = 35)	Smokers without COPD (n = 50)
Male	29	21	24	42
Females	9	11	11	8
Sex ratio (Female/Male)	0.31	0.52	0.43	0.33
Mean age (Standard)				
Male	47±13.8	44.95±16.09	45±15.08	52±13.54
Females	57.7±15.94	37.80±11.83	38±11.83	60.62±7.42
FVC (% Predicted)				
Male	54±7.9	52±6.58	94±15.74	106 ±12.35
Female	52.6±6.20	50±6.97	113.9±25.20	91.5±8.34
FEV_1_/FVC (% Predicted)				
Male	58±11.3	52.76±9.25	94±14.74	99±9.87
Female	53.5±4.92	51.45±10.68	86.9±20.94	98.37±8.61
Socio Economic Status	Poor	Poor	Poor	Poor

### Definition of the subjects

The population consisted of 155 subjects (116 males and 39 females) living in the vicinity of open –cast coal mine area at Ledo, in Assam (Lat. 27°13′–27°23′N and Long. 95°35′–96′00E).

### Diagnosis and inclusion criteria of subjects

COPD was diagnosed on the basis of medical history of exposure to coal dust, physical examination and spirometry data according to American Thoracic Society (ATS) guidelines. Inclusion criteria for COPD included the following symptoms: chronic airway symptoms and signs such as coughing, breathlessness, wheezing and chronic airway obstruction defined as the FEV_1_/FVC ratio (Forced Expiratory Volume in one Second/Forced Vital Capacity) of 70% and below and an FVC (Forced vital capacity) of 80% predicted value. Based on survey data and lung function analysis, the subjects were sorted out into the following categories: (a) Smokers with COPD (n = 38, female: male ratio  = 0.31), (b) Non-smokers with COPD (n = 32, female: male ratio  = 0. 52), (c) Healthy Non-smokers (n = 35, female: male ratio  = 0. 43), (d) Smokers without COPD (n = 50, female: male ratio  = 0.33).

### Lung function analysis

Spirometry was conducted with Spirometer (Schiller) for each subject and the values of FVC (% predicted) and FEV1/FVC (% predicted) were recorded.

### Blood Sample Collection

Blood samples were collected through health camps conducted in that area through Doctors of local PHC in collaboration with Doctors of Clinical Centre, NEIST, Jorhat. Each subject participated in the survey were examined by the doctors and recommended for blood sample collection after conducting spirometry. Blood samples were obtained only after getting subject's written consent.

### DNA extraction and PCR amplification

Genomic DNA was extracted from whole blood using a GeNei™ Whole Blood DNA extraction Kit, Bangalore Genei, India. Genomic DNA was amplified by PCR using a thermal cycler (Thermal Cycler Model 2720, Applied Biosystems, USA) in a 25 µl reaction mixture containing 1.5 mM MgCl2, 25 pM of each primer, 500 µM dNTPs, 0.1 U Taq Polymerase (MBI Fermentas, USA). The PCR conditions consisted of an initial cycle of 10 minutes at 95°C, 1 minute at 34°C and 10 seconds at 72°C. Specific GSTM1 and GSTT1 primers (Sigma) for the PCR based genotyping assays were synthesized as per the sequence given by Josef zidzik et.al. (2008)[11]. The products of multiplex PCR (GSTM1-215 bp; GSTT1-480 bp; Alb-360 bp) were separated using 3% Agarose gel (Biotechnology Grade, AMRESCO). For both GST genes, subjects were categorized as having null-type, non- null type, either null-type, both null-type, both non-null types based on the PCR amplification results.

### Statistical Analysis

The frequency of alleles and genotypes prevalence and their association between COPD and Control subjects were assessed by Chi Square and Fishers Exact test. Odds Ratio (OR) and 95% Confidence Interval (CI) along with p value were calculated to describe the association. The associations for Smoker and Non Smoker between COPD and Control were analysed by binary logistic regression analysis to find out the potential confounders. A P value <0.05 was considered as statistically significant. SPSS v9 and GraphPad – Prism 5 software's were used for all the statistical analysis.

## Results

**Figure 1:**
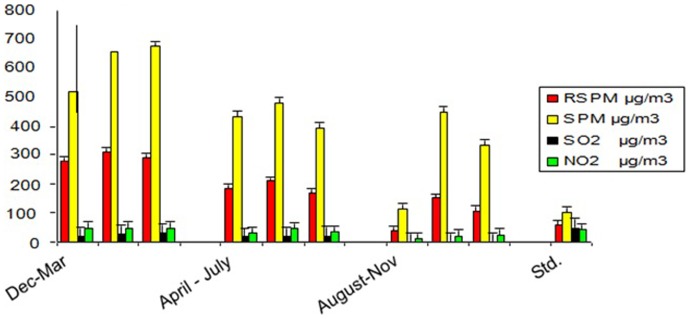
Comparative account of the concentration of respirable suspended particulate matter (RSPM), suspended particulate matter (SPM), SO_2_ and NO_2_ present in air of the coal mine site observed at different periods of the year (2012–2013) along with the standards [Variables are seasonal averages].

Air sample analysis was done for three different periods of the year- December - March, April - July and August - November. The values for Respirable suspended particulate matter (RSPM), Suspended particulate matter (SPM), SO_2_ and NO_2_ were found to be significantly higher in the study site [Figure 1]. Air samples collected from 200 kms distant area (around the institute) served as control. The highest values for RSPM, SPM, SO_2_ and NO_2_ were observed during the period December - March, followed by April - July and August –November. The values of all the parameters were found to be higher than the standard value. RSPM (29, 10, 5), SPM (41, 41, 2), SO_2_ (Trace) and NO_2_ (14, 40, 2) for control samples were within the standard limits.

**Figure 2:**
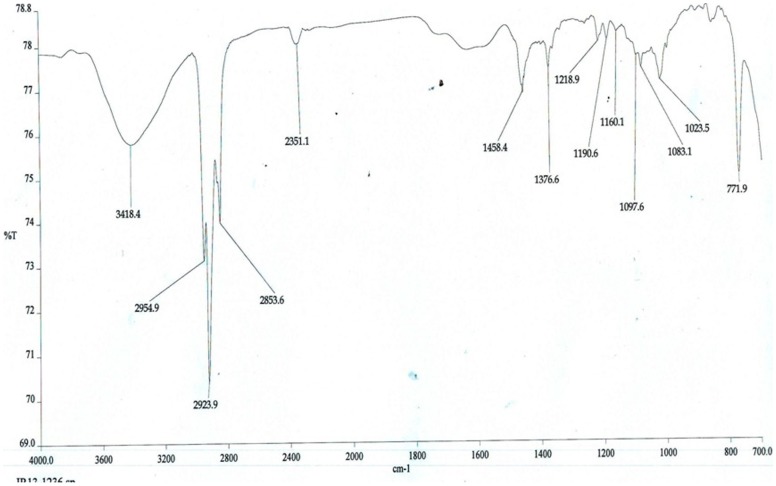
The overall FT-IR spectra of environmental dust particles present in air of coal mine site (A) collected with the help of Envirotech APM 460 DXNL respirable dust sampler, measured by Perkin Elmer 2000 FTIR spectrometer. The overall spectra from 1023.5^−1^ to 1458 cm^−1^ illustrates the concentration of major air pollutants.

**Figure 3:**
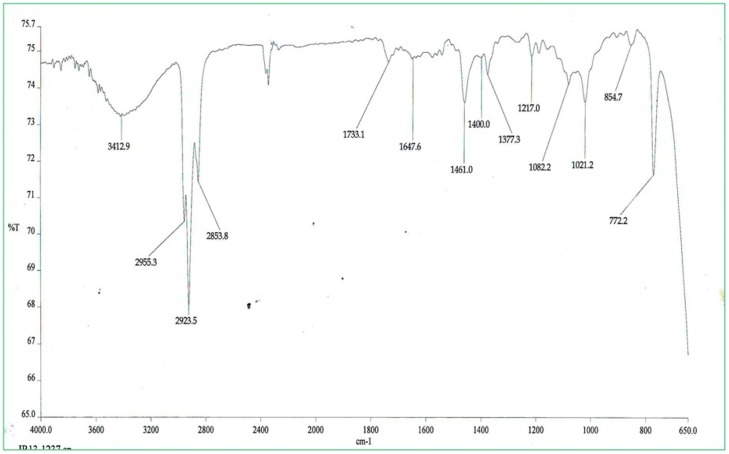
The overall FT-IR spectra of environmental dust particles present in air of the control site (B) collected with the help of Envirotech APM 460 DXNL respirable dust sampler, measured by Perkin Elmer 2000 FTIR spectrometer. The overall spectra from 1021.2^−1^ to 1461.0 cm^−1^ illustrates the major air pollutants.

The organic extracts of the coal dust samples were analysed with the help of instrument software version 4.07. The IR spectra of the dust sample (A) of coal mine site shows peaks at 1023.5 cm^−1^ and 1190.1 cm^−1^ corresponding to allopathic flora C-F stretch, 1083.1 cm-^1^ corresponding to organic silicone or silicone/aliphatic flora C-F stretch, 1217.4 cm-^1^ corresponding to aromatic C-H stretch in plane bend, whereas 1378.1 cm^−1^ and 1458.8 cm^−1^ corresponds to methyl C-H asymmetric/symmetric bending. On comparing the spectrum of dust samples of both the sites, it was observed that the sample (A) [Figure 2] shows very sharp peaks as compared to the spectrum of non - coal mine site dust sample (B) [Figure 3] which shows less intense broad peaks at 1021.2 cm^−1^, 1082.2 cm^−1^,1217.0 cm^−1^,1377.3 cm^−1^, 1400.0 cm^−1^, 1461.0 cm^−1^. The sharp peak at 1083.1 cm−1 clearly shows the higher organic silicone content in the coal mine site than the non -coal mine sites spectral peak at 1082.2 cm−1. Interpretations of the FTIR spectra were done as per John Coates [12] ^.^

For the assessment of lung function of each subject as per the spirometry results the Forced Vital Capacity (FVC) was calculated as % predicted. The smokers with COPD group and Non-smokers with COPD group showed an obstructive pattern of lung function. For smokers with COPD, FVC values were found to be 54±7.9 among males and 52.6±6.20 among females. For Non-smokers with COPD, among males the values found to be 52±6.51 and 50±6.97 among females. In case of healthy non-smokers and smokers without COPD, no lung function decline was observed. For healthy non-smokers, FVC % predicted values were 94±15.74 among males and 113.9±25.2 among females. The smokers without COPD the values were 106±12.35 among males and 91.5±8.34 among females. To find the ratio of (Forced expiratory volume in 1 second/Forced vital capacity) FEV_1_/FVC, the values were calculated as % predicted. For male smokers with COPD and non-smokers with COPD, the values were observed as 58±11.3 and 52.76±9.25 respectively. For females, the values were found to be 53.5±4.92 and 51.45±10.68 respectively. Among the healthy non-smokers and smokers without COPD the values were found to be 94±15.74 and 106±12.35 in males, 113.9±25.20 and 91.5±8.34 in females respectively. So it can be said that smoking cessation remains the single most effective intervention to reduce the risk of COPD or to slow its progression.

**Figure 4:**
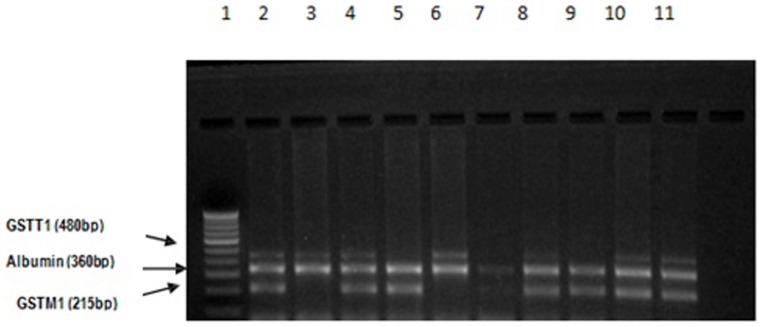
Polymorphism of GSTM1 and GSTT1 genes. Lane 1 is the molecular marker of 100

The multiplex PCR products of both the genes were of expected size [Figure 4]. Among the studied individuals, the prevalence of the GSTM1 null genotype in COPD individuals (51.4%) was observed to be almost two times higher than that found in the Control (27.1%) [Table 2]. The study showed there was a significant link between GSTM1 null genotype and COPD with p<0. 05 (OR  = 2. 9, 95% CI  = 1.5–5.6). However the prevalence of GSTT1 null genotype was not found statistically significant among COPD subjects (51.4%) and Controls (48.6%). When the proportion either null genotype of the two genes were considered we found two times higher ratio in COPD subjects compared to control. Statistical analysis by Chi square test and logistic regression analysis also reveals the same conclusion.

**Table 2:** Frequency distribution chart of GSTT1 gene and GSTM1 gene polymorphism with respect to the risk of developing COPD (** - Statistically significant P<0.05).

Genotype	Controls (n = 85)	COPD (n = 70)	P value	OR (95% CI)
GSTM1				
NON NULL	62 (72.9%)	34 (48.6%)		1.0
NULL	23 (27.1%)	36 (51.4%)	.002**	2.9 (1.5–5.6)
GSTT1				
NON NULL	50 (58.8%)	34 (48.6%)		
NULL	35 (41.2%)	36 (51.4%)	0.203	1.5 (0.8–2.9)
GSTM1+GSTT1				
BOTH NON NULL	37 (43.5%)	12 (17.1%)		1.0
BOTH NULL	15 (17.6%)	12 (17.1%)	0.077	2.5 (1.0–6.7)
EITHER NULL	33 (38.8%)	46 (65.7%)	000**	4.3 (2.0–9.5)

## Discussion

The respirable mixed toxic dust particles generally inhaled may either be due to occupational exposure to polluted environment or self-administered as cigarette smoking. This form of inhalation injury causes a low grade inflammatory exudation of fluids into both large and small bronchi, bronchioles with minimal effects on lung function but in susceptible individuals, this normal inflammatory response is amplified. In our study, we have attempted to assess the effect of environmental determinants (smoking and air pollution) on pulmonary function related to some genetic determinants (GSTM1 and GSTT1).

The air analysis (done in three different periods of the year) showed highest RSPM, SPM, SO_2_ and NO_2_ during December - March and lowest during August - November. The overall annual data reveal that our study site is considerably polluted and the population in the area is exposed to recurrent episodes of acute air pollution. Among the surveyed population, 155 subjects attended the health camp. Spirometry was done for all the subjects to analyse the lung function in smokers with COPD and non-smokers with COPD. Healthy non-smokers and smokers without COPD showed normal lung functioning. The low lung function reveals an association of air pollution with smoking history. Other than smoking habit and genetic susceptibility our study population were also under significant exposure to respirable mixed coal dust. As suggested by Love et al. [13] our FTIR studies based on the principle of sharp peak in the spectra corresponds to higher concentration, also showed a higher concentration of organic silicone or silica dust and chloro flouro compounds (1083.1 cm^−1^) present in air of coal mine site as compared to non-coal mine site (1082.2 cm^−1^). It is generally known that the open cast coal mine areas are one of the most polluted areas, but to assess the compounds which are present in higher concentration in air surrounding the coal mine sites, we have studied the air samples through infrared spectroscopy. CFC as well as the organic silicone was found to cause many respiratory diseases including silicosis. Since we employed 1.6 µm filter papers for dust sampling, so certainly the size of these dust particles may be ≤1.6 µm. These tiny particles may get embedded in the alveolar sacs triggering the immune response to these xenobiotic compounds inside the lungs ultimately leading to cellular lesions. So the organic silicone may also play a role in the development of COPD.

Cigarette smoking is well known as the major risk factor for the development of COPD in the genetically susceptible subjects [14]–[16]. To confirm our hypothesis, the subjects were genotyped for GSTM1 and GSTT1gene. Enzymatic antioxidants are under genetic control and allelic variation alters their abilities to reduce free radicals. The GSTs are important components of lung defence in response to oxidative stress and are highly polymorphic [9]. Studies in the role of polymorphism of genes regulating the GST enzyme including GSTT1, GSTM1 in reference to COPD has been conducted with controversial results in various ethnic groups and populations [16]–[18].

Several longitudinal, epidemiological and associative studies have established that acute episodes of atmospheric pollution were associated with a number of health complications [19]–[23]. A cross-sectional study in Merseyside conducted by Brabin et al. [24] in school children aged 5–11 years exposed to coal dust and air pollution established significant [Odds Ratio 1.55 (1.17–2.06) at 95% CI] increased prevalence of lung function decline irrespective of their parents being smokers or non-smokers. Leigh et al. [25] studied the relation between the emphysema and the lung coal content and found the extent of emphysema had a strong positive quantitative relation with coal content of the lungs (p<0.0003), age (p<0.0001), and smoking (p<0.0001). A Swiss study looked at the respiratory health of primary school children and noted that respiratory symptoms were highest in the area with the highest level of suspended particulates in the air and that they were independent of levels of nitrogen and sulphur oxides [26]. The coking works study from the north of England and the Munich/Leipzig study from Germany also found similar results [27]. Such form of inhalational injury causes a low grade inflammatory exudation of fluid and cells into both large and small bronchi, bronchioles with minimal effects on lung function but in susceptible individuals, this normal inflammatory response is amplified. Studies related to the role of gene polymorphism regulating the GST enzymes including GSTT1, GSTM1 in reference to COPD has been conducted with controversial results in various ethnic groups and populations [28]–[30] in non-coal mine areas.

We made an attempt to evaluate the association of GSTM1, GSTT1 and for the combination of GSTM1 & GSTT1 genotypes among the individuals of Non Smokers and Smokers irrespective of age and sex. The prevalence of GSTM1 null genotype with smoking habit was found higher i.e. 57.9% of COPD individuals compared to 31.4% in Control and was found statistically significant with a fourfold risk among the individuals with a combination of GSTM1 null genotype and smoking habit (p<0. 05, OR  = 4. 4, CI  = 1.7–10.9). Whereas in GSTT1 genotype subjects with a combination of null genotype and no habit of smoking (Non Smoker) observed statistical significant (p<0. 05, OR  = 2.8, CI  = 1.0–7.6). It was also observed that subjects with GSTT1 null genotype and Non Smoker was with a frequency of 56.3% in COPD against 31.4% in Control. In the combination of GSTM1 & GSTT1 genotypes among the individuals of Non Smoker and Smoker it was found that the individuals with a combination with either null gene and Non-smoker was in eighth fold risk (p<0. 05, OR  = 8.5, CI  = 2.3–30.9).

From the regression analysis between the smoking status and polymorphism of both the genes revealed that the smokers with GSTM1 null genotype, non-smokers with either null genotype of the two genes found to be more susceptible to COPD [Table 3 & Table 4]. It is observed that Non Smoker with null genotype of GSTT1 genotype have a threefold risk compared to Non-smoker with Non Null GSTT1 genotype (p<0.05, OR  = 2.8 CI  = 1.0–7.6), whereas in case of GSTM1 and GSTT1 genotype both together, it was found that Smoker with either Null genotype have a four time risk (p<0. 05 OR = 4. 5 CI  = 1.3–15.4).

**Table 3:** Associative studies relating to smoking and non-smoking habit to the polymorphism of GSTM1 gene and GSTT1 gene by *χ* ^2^ test (** - Statistically significant P<0.05).

Habit	Genotype	Controls (n = 85)	COPD (n = 70)	P value	OR (95% CI)
NON SMOKER	GSTM1				
	NON NULL	24 (68.6%)	18 (56.3%)		1.0
	NULL	11 (31.4%)	14 (43.8%)	0.298	1.7 (0.6–4.6)
SMOKER	GSTM1				
	NON NULL	38 (76.0%)	16 (42.1%)		1.0
	NULL	12 (24.0%)	22 (57.9%)	0.001**	4.4 (1.7–10.9)
NON SMOKER	GSTT1				
	NON NULL	24 (28.6%)	14 (43.8%)		1.0
	NULL	11 (31.4%)	18 (56.3%)	0.041**	2.8 (1.0–7.6)
SMOKER	GSTT1				
	NON NULL	26 (52.0%)	20 (52.6%)		
	NULL	24 (48.0%)	18 (47.4%)	0.953	1.0 (0.4–2.3)
NON SMOKER	GSTM1+GSTT1				
	NON NULL	17 (48.6%)	4 (12.5%)		1.0
	EITHER NULL	12 (34.3%)	24 (75.0%)	000**	8.5 (2.3–30.9)
	BOTH NULL	6 (17.1%)	4 (12.5%)	0.381	2.8 (0.5–15.1)
SMOKER	GSTM1+GSTT1				
	NON NULL	20 (40.0%)	8 (21.1%)		1.0
	EITHER NULL	21 (42.0%)	22 (57.9%)	0.060	2.6 (1.0–7.2)
	BOTH NULL	9 (18.0%)	8 (21.1%)	0.209	2.2 (0.6–7.8)

**Table 4:** Associative studies relating to smoking and non-smoking habit with the polymorphism of GSTM1 and GSTT1 genes by binary logistic regression analysis (Variables taken were habit status and Null/Non Null genotype of both the genes) (** Statistically significant P<0.05).

Genotype	Controls (n = 85)	COPD (n = 70)	P value	OR (95% CI)
GSTM1				
NON SMOKER + NON NULL	24 (28.2%)	18 (25.7%)		1.0
SMOKER + NON NULL	38 (44.7%)	16 (22.9%)	0.181	0.6 (0.2–1.3)
NON SMOKER + NULL	11 (12.9%)	14 (20.0%)	0.299	1.7 (0.6–4.6)
SMOKER + NULL	12 (14.1%)	22 (31.4%)	0.060	2.4 (1.0–6.2)
GSTT1				
NON SMOKER + NON NULL	24 (28.2%)	14 (20.0%)		1.0
SMOKER + NON NULL	26 (30.6%)	20 (28.6%)	0.538	1.3 (0.5–3.2)
NON SMOKER + NULL	11 (12.9%)	18 (25.7%)	0.043**	2.8 (1.0–7.6)
SMOKER + NULL	24 (28.2%)	18 (25.7%)	0.584	1.3 (0.5–3.2)
GSTM1+GSTT1				
NON SMOKER+BOTH NON NULL	17 (20.0%)	4 (5.7%)		1.0
NON SMOKER+ EITHER NULL	12 (14.1%)	24 (34.3%)	.001**	8.5 (2.3–31.0)
NON SMOKER+ BOTH NULL	6 (7.1%)	4 (5.7%)	.221	2.8 (0.5–15.0)
SMOKER+ BOTH NON NULL	20 (23.5%)	8 (11.4%)	.446	1.7 (0.4–6.6)
SMOKER + EITHER NULL	21 (24.7%)	22 (31.4%)	.018**	4.5 (1.3–15.4)
SMOKER + BOTH NULL	9 (10.6%)	8 (11.4%)	.072	3.8 (0.9–16.1)

From the study it is revealed that among the studied subjects prevalence of the GSTM1 null genotype and for combined GSTM1 & GSTT1 either null genotype in COPD individuals was higher than that found in Control. The study showed there was a significant link between GSTM1 null genotype and for combined GSTM1 & GSTT1 either null genotype as compared with COPD.

It is important to note that the study population were exposed to the respirable mixed coal dust. The analysis of the parameters was done during the period January 2012 to January 2013 and no seasonal variation was reported on the lung function of the subjects. There was no data available on the status of GST gene with reference to COPD subjects staying near the open-cast coal mine at Ledo, Assam. Further, the ethnicity (migrant labour) of the people of this region is very different from rest of the country. Barnes et al. [31] mentioned that differences in the prevalence of COPD in different ethnic groups are likely to be accounted by the different frequencies of genes relevant to pathogenesis, so that exploration of these differences at different molecular level may be informative. Although Yim et al. [32], He et al. [33], Cahkoglu et al. [34], Lakhdar et al. [35], have extensively studied the association of GSTM1 null genotype with the severity of COPD, but they have not found any correlation between the null genotype and COPD. In contrast, in the present study we have evaluated that the smokers with GSTM1 (null genotype) were more susceptible to COPD with additional contribution of respirable coal dust from the coal mine. However, Baranova et al. also suggested the homozygous deletion of GSTM1gene is associated with complete loss of enzyme production and is associated with severe chronic bronchitis in heavy smokers [32].

The limitations of the study were lack of age and sex matching between all the groups. We evaluated the effect of smoking history and GSTM1 (null) genotype in the pathogenesis of COPD.

Thus we can conclude that the GSTM1 (null genotype) appears to be a risk factor for the low lung function in smokers living in the vicinity of open cast coal mine area and the presence of at least one active allele (either GSTM1/GSTT1) seemed to have a protective role in the development of COPD. Further studies with the association of HMOX1 and MMP12 gene, will be necessary to elucidate the pathogenesis of COPD as a genetic disease in compliance with environment with larger sample size.
